# Conversion surgery for stage IV gastric cancer with a complete pathological response to nivolumab: a case report

**DOI:** 10.1186/s12957-020-01954-0

**Published:** 2020-07-21

**Authors:** Ryu Matsumoto, Takaaki Arigami, Daisuke Matsushita, Keishi Okubo, Takako Tanaka, Shigehiro Yanagita, Ken Sasaki, Masahiro Noda, Yoshiaki Kita, Shinichiro Mori, Hiroshi Kurahara, Takao Ohtsuka

**Affiliations:** 1 Department of Digestive Surgery, Breast and Thyroid Surgery, Kagoshima University Graduate School of Medical and Dental Sciences, Kagoshima, Japan; 2 Department of Onco-biological Surgery, Kagoshima University Graduate School of Medical and Dental Sciences, 8-35-1 Sakuragaoka, Kagoshima, 890-8520 Japan

**Keywords:** Conversion surgery, Nivolumab, Responder, R0 resection, Unresectable gastric cancer

## Abstract

**Background:**

Patients with stage IV gastric cancer have a poor prognosis despite the recent development of multidisciplinary treatments that include chemotherapy. However, conversion surgery has emerged as a promising strategy to improve the prognosis in responders with unresectable gastric cancer after chemotherapy. Moreover, nivolumab is currently recommended as a third-line treatment in patients with unresectable advanced gastric cancer. However, there are few reports of conversion surgery after nivolumab in patients with stage IV gastric cancer.

**Case presentation:**

A 68-year-old woman complaining of nausea was diagnosed with stage I gastric cancer (T2N0M0). Although we planned gastrectomy with lymphadenectomy, multiple liver metastases were detected during the surgery. After staging laparoscopy, we diagnosed this patient as having stage IV unresectable gastric cancer, and we administered chemotherapy and immunotherapy for 39 months (first-line regimen: 6 courses of S-1 plus oxaliplatin; second-line regimen: 6 courses of ramucirumab plus paclitaxel; and third-line regimen: 20 courses of nivolumab). Although the liver metastases completely disappeared after the second-line chemotherapy, lung metastases and a rapid enlargement of the primary tumor were confirmed. Consequently, the patient received nivolumab at a dose of 3 mg/kg intravenously every 2 weeks, then a dose of 240 mg/kg intravenously every 2 weeks from September 2018. After 20 courses of nivolumab, the primary tumor dramatically shrank and the lung metastases disappeared. The patient had a partial primary tumor response to nivolumab. Therefore, the patient underwent laparoscopic distal gastrectomy with D2 lymph node dissection. The macroscopic examination of the resected specimen showed an ulcer scar in the primary tumor site. The pathological examination demonstrated no residual tumors and no lymph node metastases, and the histological response of the primary tumor was categorized as grade 3. The postoperative course was uneventful, and the patient is receiving nivolumab to control potential liver and lung metastases.

**Conclusions:**

Conversion surgery might help control tumor progression in responders after chemotherapy and immunotherapy.

## Background

Gastric cancer is the fifth most common malignancy and the second leading cause of cancer-related deaths worldwide, with 1,033,700 cases and 782,700 deaths recorded in 2018 [1]. Furthermore, the prognosis of patients with stage IV gastric cancer is poor despite the remarkable development of multidisciplinary treatments that include chemotherapy: the median overall survival (OS) of patients with stage IV gastric cancer is 9–11 months [2]. On the other hand, immune checkpoint blockade has emerged as a novel immune therapy for patients with various malignant neoplasms, including gastric cancer [3, 4]. A recent study demonstrated that nivolumab, an anti-programmed cell death protein 1 antibody, had offered clinical prognostic improvements in patients previously treated with advanced gastric or gastroesophageal junction cancer [5, 6]. Currently, the Japanese Gastric Cancer Treatment Guidelines 2018 recommends nivolumab as the third-line chemotherapy in patients with unresectable advanced or recurrent gastric cancer [7].

Recently, conversion surgery has emerged as a promising therapeutic tool for providing long-term survival in responders with stage IV gastric cancer after chemotherapy [8, 9]. However, there are few reports regarding the surgical intervention of patients with multiple metastatic lesions, such as both liver and lung metastases from gastric cancer [10, 11]. Additionally, the clinical indication and prognostic significance of conversion surgery remains unclear in responders after nivolumab. Herein, we present a case of stage IV gastric cancer showing a pathological complete response by laparoscopic conversion surgery after chemotherapy and immunotherapy.

## Case presentation

**Fig. 1:**
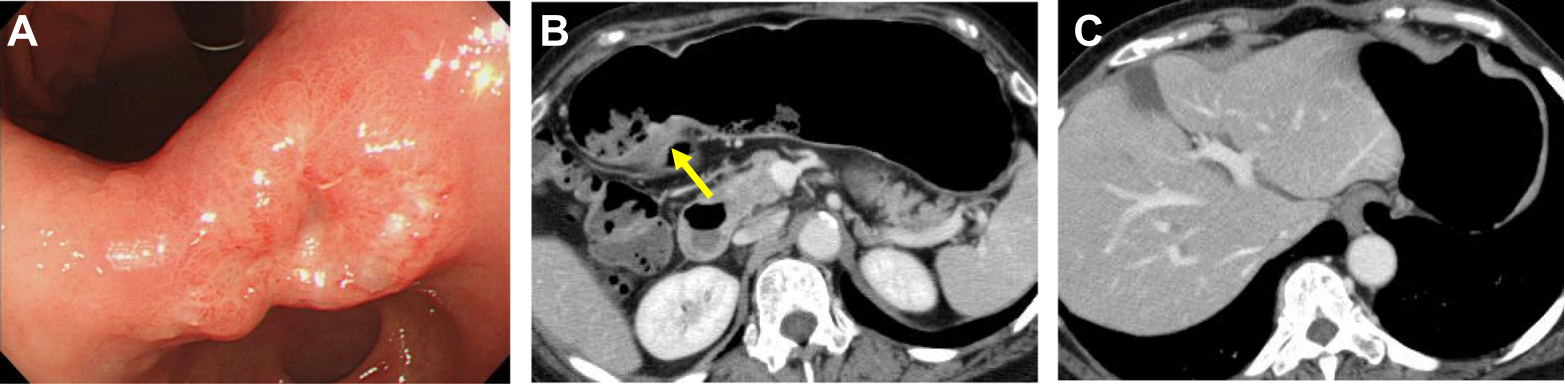
**a** Esophagogastroduodenoscopy revealed an irregular and nodular tumor on the middle third region of the stomach. **b** Computed tomography (CT) showed thickening of the gastric wall in tumor site (arrow). **c** No lymph node metastasis or distant metastasis was detected by CT

**Fig. 2:**
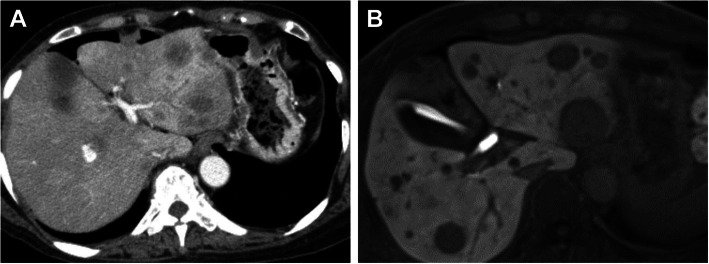
**a** Computed tomography showed multiple liver metastases in the both liver lobes. **b** Magnetic resonance imaging

**Fig. 3:**
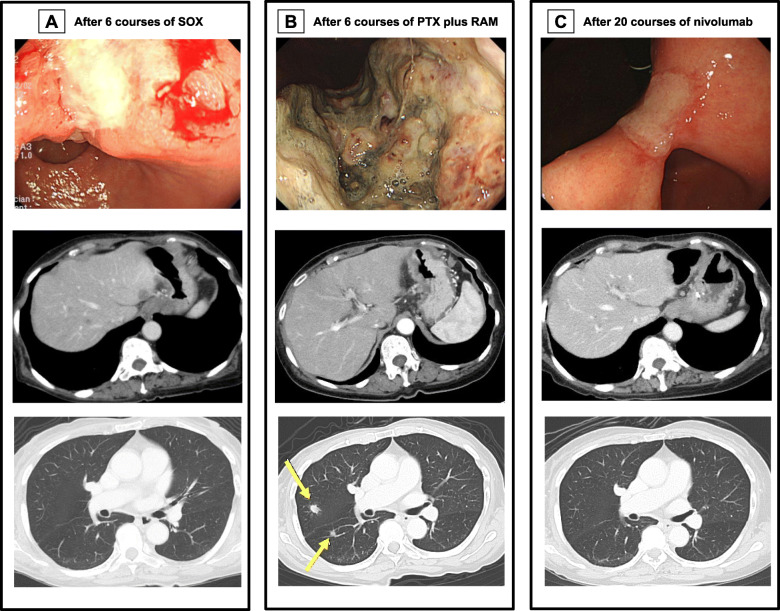
**a** After 6 courses of S-1 plus oxaliplatin. **b** After 6 courses of paclitaxel plus ramucirumab. Computed tomography showed lung metastases (arrows). **c** After 20 courses of nivolumab

**Fig. 4:**
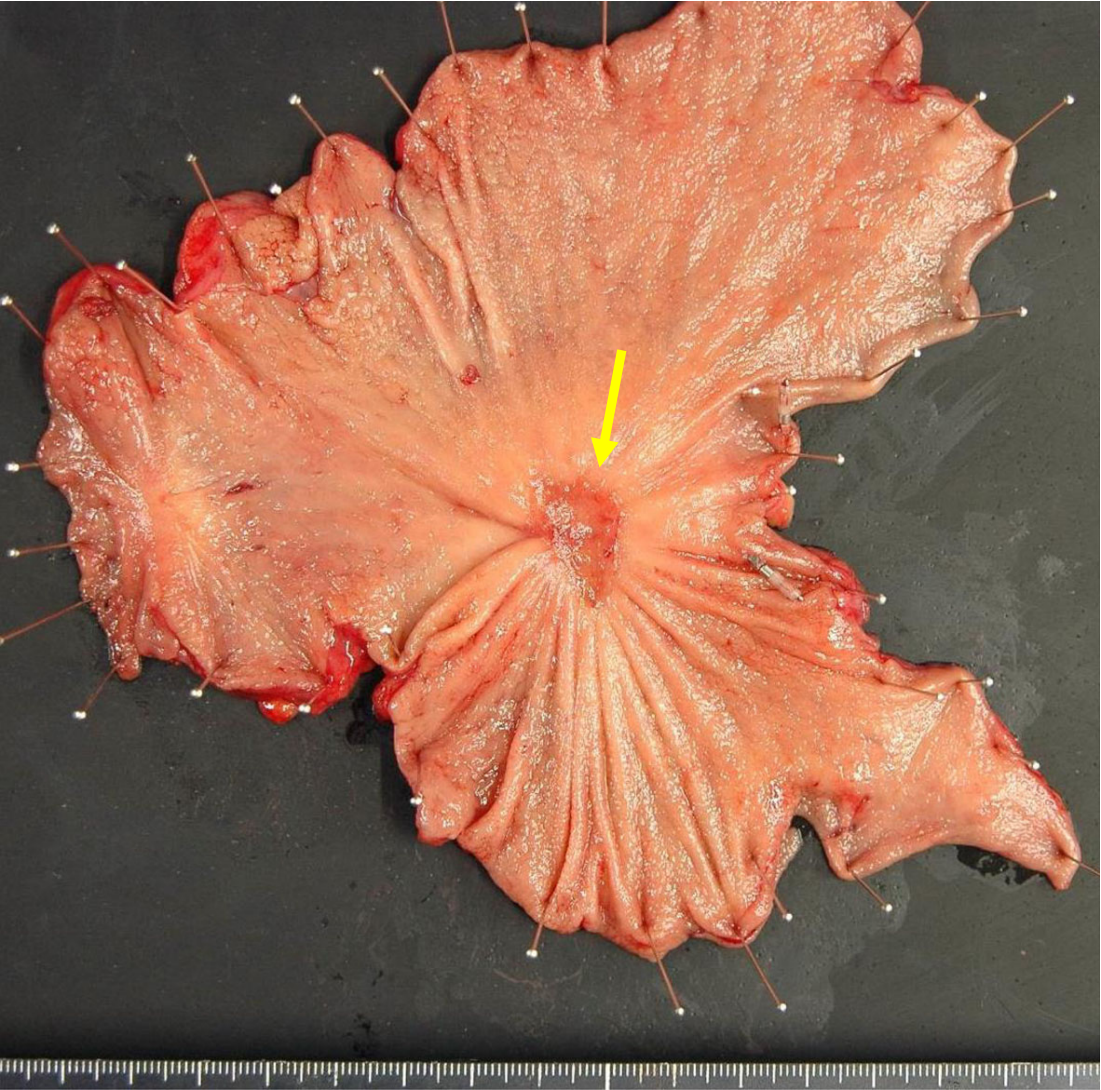
Macroscopic findings of the resected stomach. An ulcer scar was shown in primary tumor site (arrow)

**Fig. 5:**
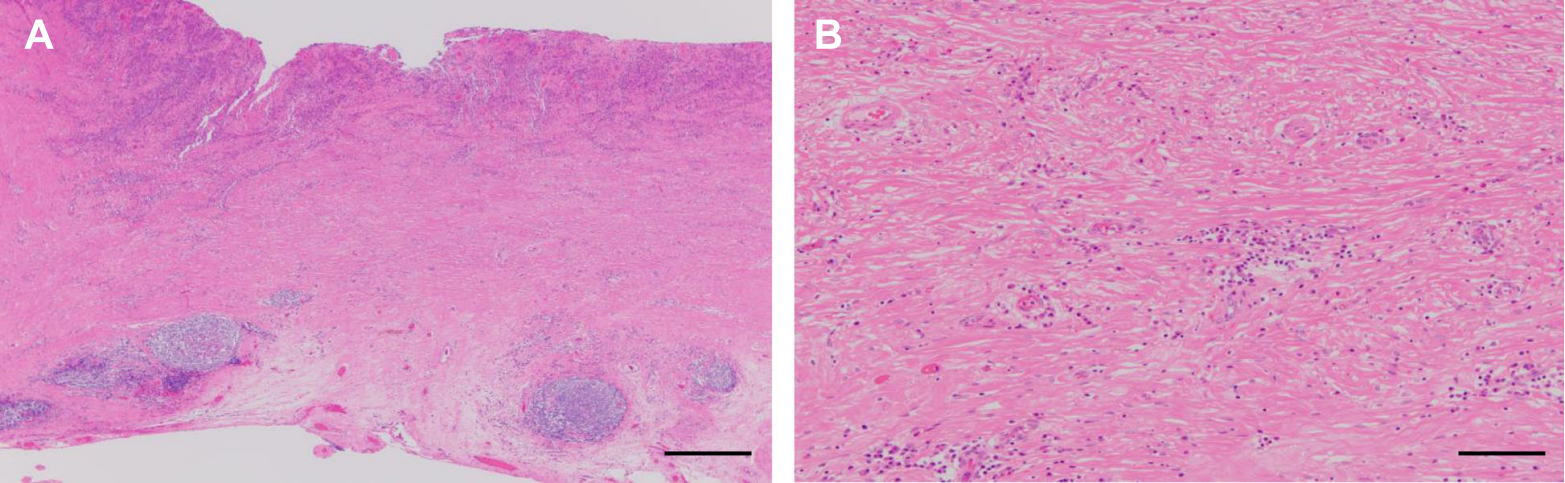
Hematoxylin-eosin staining. **a** The scale bar indicates 500 μm (×40 magnification). **b** The scale bar indicates 100 μm (×200 magnification). The pathological examination showed an ulcer scar with infiltrating lymphocytes and plasma cells without residual tumors

A 68-year-old woman complaining of nausea was diagnosed with early gastric cancer at her local hospital and was referred to us for further evaluation. The patient had an Eastern Cooperative Oncology Group Performance Status (ECOG PS) of 1. Her serum levels of carcinoembryonic antigen (CEA) and carbohydrate antigen (CA) 19-9 were 11.7 ng/ml and 317.9 U/ml, respectively. Esophagogastroduodenoscopy (EGD) revealed an irregular and nodular tumor with ulcerative changes in the middle third region of the stomach (Fig. 1a). Endoscopic ultrasonography showed that the tumor had possibly invaded the muscularis propria. The pathological examination of the biopsied specimen indicated a poorly differentiated adenocarcinoma. Although enhanced computed tomography (CT) showed a thickening of the gastric wall in the tumor site, no lymph node metastasis or distant metastasis was identified (Fig. 1b, c). Consequently, the patient was clinically diagnosed with stage I gastric cancer (T2N0M0). One month after the first visit, we planned laparoscopic distal gastrectomy with D2 lymphadenectomy. However, multiple liver metastases were identified during the surgery. Therefore, we suspended curative gastrectomy, and chemotherapy was selected due to her satisfactory ECOG PS. After staging laparoscopy, enhanced CT and magnetic resonance imaging revealed multiple liver metastases in both liver lobes (Fig. 2a, b). Given the patient was diagnosed with human epidermal growth factor receptor 2-negative stage IV gastric cancer, the patient received S-1 plus oxaliplatin (SOX) as first-line chemotherapy. The SOX regimen consisted of a 3-week course of S-1 (80 mg/body/day) orally on days 1–14, with oxaliplatin (130 mg/m^2^) intravenously on day 1. After 6 courses of the SOX regimen, the liver metastases were substantially reduced and new metastatic lesions were not detected (Fig. 3a). However, the patient had progression in the primary tumor site (Fig. 3a). Therefore, we changed the chemotherapy regimen to paclitaxel (PTX) plus ramucirumab (RAM) as the second-line chemotherapy. This regimen consisted of a 4-week course of PTX (80 mg/m^2^) intravenously on days 1, 8, and 15, with RAM (8 mg/kg) intravenously on days 1 and 15. After 6 courses of PTX plus RAM, enhanced CT indicated that the liver metastases had completely disappeared (Fig. 3b). However, EGD and enhanced CT showed enlargement of the primary tumor and lung metastases, respectively (Fig. 3b). According to the Response Evaluation Criteria in Solid Tumors (RECIST) [12], the patient had progressive disease. Accordingly, nivolumab was administered as the third-line chemotherapy. The patient received nivolumab at a dose of 3 mg/kg intravenously every 2 weeks and a dose of 240 mg/body intravenously every 2 weeks after September 2018. After 20 courses of nivolumab, EGD and enhanced CT revealed a dramatic shrinkage of the primary tumor and the disappearance of the lung metastases, respectively (Fig. 3c). Fluorodeoxyglucose positron emission tomography showed no abnormal uptake. Furthermore, the patient’s serum levels of CEA and CA 19-9 were within normal limits (CEA: 2.6 ng/ml and CA19-9: 8.3 U/ml). Given the patient had partial response based on the RECIST criteria [12], a surgical intervention was planned. The staging laparoscopy indicated no peritoneal dissemination and negative peritoneal cytology; thus, the patient underwent laparoscopic distal gastrectomy with D2 lymphadenectomy. The macroscopic findings of the resected specimen showed an ulcer scar at the primary tumor site (Fig. 4). The pathological examination demonstrated that there were no residual tumors and no lymph node metastases (Fig. 5). These findings indicated a pathological complete response, and the histological response of the primary tumor was categorized as grade 3. The postoperative course was uneventful, and the patient was discharged home on the eighth postoperative day. Currently, the patient is receiving nivolumab to control potential liver and lung metastases. The patient is alive at 41 months after the first-line chemotherapy, with no sign of disease recurrence at 3 months post-surgery.

## Discussion and conclusions

We report a case of stage IV gastric cancer that was successfully treated with chemotherapy, immunotherapy, and subsequent conversion surgery. Moreover, the patient had a pathological complete response to nivolumab. To the best of our knowledge, we are the first to describe a case report of stage IV gastric cancer showing a complete pathological response to nivolumab.

The Japanese Gastric Cancer Treatment Guidelines of 2018 recommend systemic chemotherapy for patients with unresectable advanced and recurrent gastric cancer, an ECOG PS 0–2, and preserved major organ function [7]. The recent development of chemotherapy combined with molecular targeted drugs, such as trastuzumab and ramucirumab, has contributed to an improved prognosis in patients with stage IV gastric cancer [13, 14]. Although the present case showed the shrinkage of multiple liver metastases after the first-line chemotherapy, the primary tumor markedly increased. Accordingly, surgical interventions after chemotherapy offer the clinical benefit of removing residual tumors in patients with unresectable advanced and recurrent gastric cancer.

In recent years, immune checkpoint blockade has been introduced in the clinical management of patients with unresectable advanced and recurrent gastric cancer [5, 6]. Nivolumab is a representative immune checkpoint blockade, and our patient received it as third-line chemotherapy. The ATTRACTION-2 trial demonstrated that the 2-year OS rates were 10.6% and 3.2% in the nivolumab and placebo groups, respectively [6]. The 2-year update data indicated that the objective response rates were 11.9% and 0% in the nivolumab and placebo groups, respectively [6]. Interestingly, several authors have reported that the inhibition of vascular endothelial growth factor signals changes in the tumor environment, which might have influenced the efficacy of nivolumab [15, 16]. Kato et al. had reported that the disease control rates were 55.6% and 42.2% in the nivolumab with and without previous RAM treatments, respectively [16]. Additionally, better clinical effectiveness was observed in patients who received nivolumab directly after RAM treatments [16]. The present patient received nivolumab after RAM treatments as third-line chemotherapy, after which the primary tumor shrank and the lung metastases disappeared. These findings suggest that nivolumab after RAM treatment is an effective tool in the therapeutic strategy of chemotherapy.

Recent studies have demonstrated the prognostic impact of conversion surgery after chemotherapy in various malignancies, including gastric cancer [8, 9, 17, 18]. Yoshida et al. proposed a new biological classification for the optimal therapeutic strategy for patients with stage IV gastric cancer and indicated conversion surgery after chemotherapy [8]. However, the clinical significance of conversion surgery remains unclear in patients with stage IV gastric cancer. Boem et al. had reported that the median survival time (MST) was 26.0 months in a retrospective study of 101 patients with stage IV gastric cancer undergoing conversion surgery after chemotherapy [9]. They concluded that conversion surgery had a prognostic benefit in selected patients who showed a good clinical response to chemotherapy [9]. In the clinical management for conversion surgery, there has been increasing attention on chemotherapy regimens, the optimal time for surgery, the selection criteria, and the indication for complete surgical resection [8, 19]. In Japan, chemotherapy regimens are determined by the 2018 Japanese Gastric Cancer Treatment Guidelines [7]. When tumors have a good response to chemotherapy, conversion surgery would be planned for curative resection with negative resection margins (R0). In the present case, we selected chemotherapy regimens for the first-line, second-line, and third-line treatments based on the Japanese guidelines. Given that primary tumor shrinkage and disappearance of liver and lung metastases were shown after 20 courses of nivolumab, conversion surgery was planned. Moreover, no peritoneal dissemination or negative peritoneal cytology was observed via staging laparoscopy. Therefore, the present case underwent conversion surgery and R0 resection. According to the Japanese classification of gastric carcinoma, the presence or absence of residual tumor after surgery is defined as an R status [20]. Several studies have demonstrated that an R status is one of the most important prognostic factors in patients with advanced gastric cancer undergoing conversion surgery [21–24]. Yamaguchi et al. had reported that the MST was 41.3 months and 21.2 months in patients with R0 and R1-2 resection undergoing conversion surgery after chemotherapy, respectively [21]. Consequently, these findings indicate the prognostic impact of the R status in conversion surgery for advanced gastric cancer.

Surprisingly, the present case had a pathological complete response, and grade 3 was shown in the histological response of the primary tumor. Generally, a pathological complete response is a rare phenomenon in the chemotherapeutic management of patients with gastric cancer [25]. Furthermore, the ATTRACTION-2 trial showed that complete response rate was 1.1% in the overall population [6]. However, the pathological examination showed an ulcer scar with infiltrating lymphocytes and plasma cells at the primary tumor site of the present case. Cho et al. reported that the 5-year OS and recurrence-free survival rates were 85% and 75%, respectively, in patients with advanced gastric cancer who achieved a pathological complete response with neoadjuvant chemotherapy [25]. These findings propose that patients with a pathological complete response have a favorable prognosis in gastric cancer. Consequently, the patients in the present case might be expected to have a promising long-term survival.

In conclusion, we have presented a case with stage IV gastric cancer and a confirmed pathological complete response by laparoscopic conversion surgery after immunotherapy. We suggest that conversion surgery might have clinical utility for controlling tumor progression in responders with a curative R0 resection after immunotherapy.

## Data Availability

The datasets obtained during the current study are available from the corresponding author on reasonable request.

## Abbreviations

CA: Carbohydrate antigen
CEA: Carcinoembryonic antigen
CT: Computed tomography
ECOG PS: Eastern Cooperative Oncology Group Performance Status
EGD: Esophagogastroduodenoscopy
MST: Median survival time
OS: Overall survival
PTX: Paclitaxel
RAM: Ramucirumab
RECIST: Response Evaluation Criteria in Solid Tumors
SOX: S-1 plus oxaliplatin
